# Poly[[μ_4_-bis­(4-pyridyl­carbon­yl)piperazine-κ^4^ *N*:*N*′:*O*:*O*′]bis­(thio­cyanato-κ*N*)cobalt(II)]

**DOI:** 10.1107/S160053681001915X

**Published:** 2010-05-29

**Authors:** Zachary M. Wilseck, Robert L. LaDuca

**Affiliations:** a Lyman Briggs College, Department of Chemistry, Michigan State University, East Lansing, MI 48825, USA

## Abstract

In the title compound, [Co(NCS)_2_(C_16_H_16_N_4_O_2_)]_*n*_, the octa­hedrally coordinated Co^II^ ion lies on a crystallographic inversion center, with *trans* isothio­cyanate ligands. Pyridyl N-donor atoms and formyl O-donor atoms from exotetra­dentate bis­(4-pyridyl­carbon­yl)piperazine (4-bpfp) ligands link the Co(NCS)_2_ units into a [Co(NCS)_2_(4-bpfp)]_*n*_ coordination polymer layer that is oriented parallel to (101). The layers stack along [010] to construct the pseudo-three-dimensional structure.

## Related literature

For divalent metal isophthalate coordination polymers containing bis­(4-pyridylmeth­yl)piperazine ligands, see: Martin *et al.* (2007 ▶). For a cobalt isothio­cyanate coordination polymer containing bis­(4-pyridylmeth­yl)piperazine ligands, see: Martin *et al.* (2009 ▶). For the preparation of 4-bpfp, see: Hou *et al.* (2003 ▶).
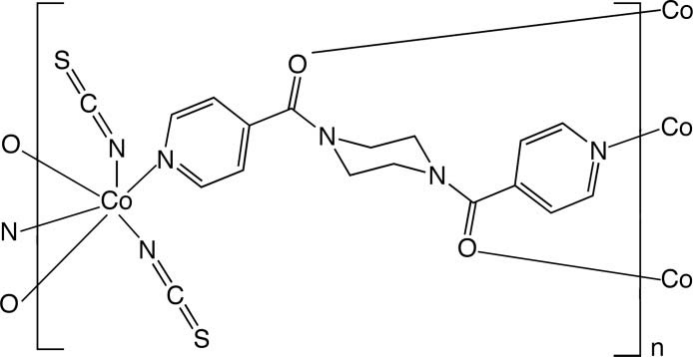


## Experimental

### 

#### Crystal data

[Co(NCS)_2_(C_16_H_16_N_4_O_2_)]
*M* *_r_* = 471.44
Triclinic, 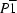

*a* = 7.7410 (14) Å
*b* = 7.8943 (15) Å
*c* = 9.801 (3) Å
α = 101.080 (2)°
β = 102.264 (2)°
γ = 119.136 (2)°
*V* = 479.58 (18) Å^3^
*Z* = 1
Mo *K*α radiation
μ = 1.14 mm^−1^
*T* = 173 K
0.42 × 0.19 × 0.07 mm

#### Data collection

Bruker APEXII CCD diffractometer
Absorption correction: multi-scan (*SADABS*; Sheldrick, 1996 ▶) *T*_min_ = 0.643, *T*_max_ = 0.928
6955 measured reflections
1753 independent reflections
1646 reflections with *I* > 2σ(*I*)
*R*_int_ = 0.052

#### Refinement

*R*[*F* ^2^ > 2σ(*F* ^2^)] = 0.037
*wR*(*F* ^2^) = 0.105
*S* = 1.18
1753 reflections
133 parameters
H-atom parameters constrained
Δρ_max_ = 0.37 e Å^−3^
Δρ_min_ = −0.55 e Å^−3^

### 

Data collection: *APEX2* (Bruker, 2007 ▶); cell refinement: *SAINT* (Bruker, 2007 ▶); data reduction: *SAINT*; program(s) used to solve structure: *SHELXS97* (Sheldrick, 2008 ▶); program(s) used to refine structure: *SHELXL97* (Sheldrick, 2008 ▶); molecular graphics: *CrystalMaker* (Palmer, 2007 ▶); software used to prepare material for publication: *SHELXL97*.

## Supplementary Material

Crystal structure: contains datablocks I, global. DOI: 10.1107/S160053681001915X/hy2308sup1.cif

Structure factors: contains datablocks I. DOI: 10.1107/S160053681001915X/hy2308Isup2.hkl

Additional supplementary materials:  crystallographic information; 3D view; checkCIF report

## Figures and Tables

**Table 1:** Selected bond lengths (Å)

Co1—N1	2.1739 (19)
Co1—N3	2.026 (2)
Co1—O1^i^	2.2034 (16)

Symmetry code: (i) 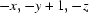.

**Fig. 1.**
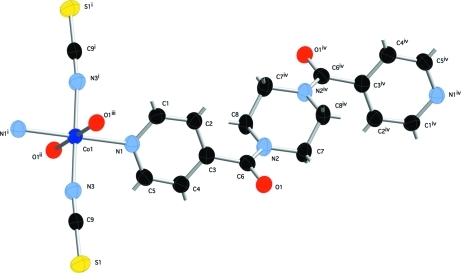
The coordination environment of the title compound, showing 50% probability ellipsoids. Hydrogen atom positions are shown as grey sticks. [Color codes: dark blue Co, yellow S, red O, light blue N, black C. Symmetry codes: (i) -x+1, -y+1, -z; (ii) -x, -y+1, -z; (iii) x+1, y, z; (iv) -x-1, -y+1, -z-1.]

**Fig. 2.**
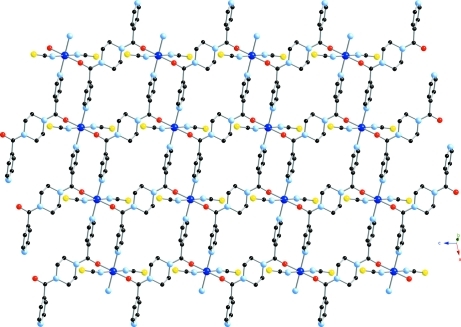
[Co(NCS)2(4-bpfp)]n layer in the title compound.

**Fig. 3.**
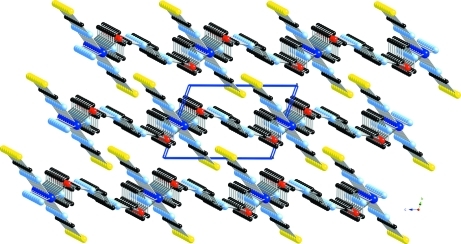
Packing diagram of the title compound.

## supplementary crystallographic information

### Comment

Recently our group has been investigating the synthesis of divalent metal coordination polymers containing aromatic dicarboxylate and bis(4-pyridyl-methyl)piperazine ligands (Martin *et al.*, 2007). To probe the structural effect of the presence of hydrogen-bond accepting formyl oxygen atoms in a similar ligand, we attempted to prepare a cobalt phthalate coordination polymer containing bis(4-pyridylcarbonyl)piperazine (4-bpfp). Use of cobalt(II) thiocyanate as the metal precursor afforded pink plates of the title compound.

The title compound crystallizes in the centrosymmetric triclinic space group with an asymmetric unit consisting of a Co^II^ ion on a crystallographic inversion center, one isothiocyanate ligand bound *via* its N atom, and one-half of a 4-bpfp molecule. The coordination environment at Co is a slightly distorted [CoN_4_O_2_] octahedron (Fig. 1), with *trans* isothiocyanate ligands, *trans* pyridyl N atom donors from two 4-bpfp ligands, and *trans* formyl O atom donors from two other 4-bpfp ligands.

Each 4-bpfp ligand is exotetradentate, ligating to Co atoms through both pyridyl N atoms and both formyl O atoms. As a result, [Co(NCS)_2_(4-bpfp)]_n_ coordination polymer layers are formed (Fig. 2), which are oriented parallel to the *ac* crystal planes. Fourteen-membered [CoOC_4_N]_2_ circuits, whose centroids rest on crystallographic inversion centers, are evident within the layer motifs. The Co···Co distances across these circuits denote the *a* lattice parameter. The through-ligand Co···Co distances across the full span of the 4-bpfp ligands measure 16.471 (4) Å.

Adjacent [Co(NCS)_2_(4-bpfp)]_n_ layers stack in an *AAA* pattern along the *b* direction (Fig. 3), with the isothiocyanate ligands projecting above and below the layer planes. Crystal packing forces cause aggregation of the layer motifs into *pseudo* three-dimensional crystal structure of the title compound.

### Experimental

All starting materials were obtained commercially, except for 4-bpfp, which was prepared by a published procedure (Hou *et al.*, 2003). Cobalt(II) thiocyanate (130 mg, 0.74 mmol), phthalic acid (123 mg, 0.74 mmol) and 4-bpfp (110 mg, 0.37 mmol) were placed into 10 ml H_2_O in a 23 ml Teflon-lined Parr acid digestion bomb. The bomb was heated at 393 K for 48 h and was then allowed to cool to room temperature. Pink plates of the title compound were obtained along with a white powdery solid.

### Refinement

All H atoms bound to C atoms were placed in calculated positions, with C—H = 0.93 (CH) and 0.97 (CH_2_) Å, and refined in a riding mode with *U*_iso_(H) = 1.2*U*_eq_(C).

### Crystal data

**Table:**

[Co(NCS)_2_(C_16_H_16_N_4_O_2_)]	*Z* = 1
*M**_r_* = 471.44	*F*(000) = 241
Triclinic, *P*1	*D*_x_ = 1.632 Mg m^−^^3^
Hall symbol: -P 1	Mo *K*α radiation, λ = 0.71073 Å
*a* = 7.7410 (14) Å	Cell parameters from 6955 reflections
*b* = 7.8943 (15) Å	θ = 2.3–25.3°
*c* = 9.801 (3) Å	µ = 1.14 mm^−^^1^
α = 101.080 (2)°	*T* = 173 K
β = 102.264 (2)°	Plate, pink
γ = 119.136 (2)°	0.42 × 0.19 × 0.07 mm
*V* = 479.58 (18) Å^3^	

### Data collection

**Table:**

Bruker APEXII CCD diffractometer	1753 independent reflections
Radiation source: fine-focus sealed tube	1646 reflections with *I* > 2σ(*I*)
graphite	*R*_int_ = 0.052
ω and φ scans	θ_max_ = 25.3°, θ_min_ = 2.3°
Absorption correction: multi-scan (*SADABS*; Sheldrick, 1996)	*h* = −9→9
*T*_min_ = 0.643, *T*_max_ = 0.928	*k* = −9→9
6955 measured reflections	*l* = −11→11

### Refinement

**Table:**

Refinement on *F*^2^	Primary atom site location: structure-invariant direct methods
Least-squares matrix: full	Secondary atom site location: difference Fourier map
*R*[*F*^2^ > 2σ(*F*^2^)] = 0.037	Hydrogen site location: inferred from neighbouring sites
*wR*(*F*^2^) = 0.105	H-atom parameters constrained
*S* = 1.18	*w* = 1/[σ^2^(*F*_o_^2^) + (0.0458*P*)^2^ + 0.1032*P*] where *P* = (*F*_o_^2^ + 2*F*_c_^2^)/3
1753 reflections	(Δ/σ)_max_ < 0.001
133 parameters	Δρ_max_ = 0.37 e Å^−^^3^
0 restraints	Δρ_min_ = −0.55 e Å^−^^3^

### Fractional atomic coordinates and isotropic or equivalent isotropic displacement parameters (Å^2^)

**Table:**

	*x*	*y*	*z*	*U*_iso_*/*U*_eq_	
Co1	0.5000	0.5000	0.0000	0.03453 (19)	
S1	0.75373 (12)	1.06782 (11)	0.44195 (8)	0.0504 (2)	
N1	0.2531 (3)	0.5500 (3)	−0.0887 (2)	0.0359 (4)	
N2	−0.3493 (3)	0.6090 (3)	−0.3539 (2)	0.0384 (5)	
N3	0.6324 (3)	0.7503 (3)	0.1850 (2)	0.0409 (5)	
C1	0.0560 (4)	0.3911 (4)	−0.1764 (3)	0.0391 (5)	
H1	0.0304	0.2594	−0.2075	0.047*	
C2	−0.1098 (4)	0.4140 (4)	−0.2225 (3)	0.0389 (5)	
H2	−0.2440	0.2996	−0.2828	0.047*	
C3	−0.0749 (4)	0.6093 (4)	−0.1781 (2)	0.0364 (5)	
C4	0.1289 (4)	0.7751 (4)	−0.0883 (3)	0.0402 (5)	
H4	0.1589	0.9085	−0.0564	0.048*	
C5	0.2849 (4)	0.7384 (4)	−0.0476 (3)	0.0402 (5)	
H5	0.4208	0.8506	0.0119	0.048*	
C6	−0.2526 (4)	0.6408 (3)	−0.2131 (3)	0.0366 (5)	
C7	−0.5284 (4)	0.6312 (4)	−0.3955 (3)	0.0418 (6)	
H7A	−0.4879	0.7492	−0.4281	0.050*	
H7B	−0.5682	0.6550	−0.3099	0.050*	
C8	−0.2853 (4)	0.5629 (4)	−0.4802 (3)	0.0407 (6)	
H8A	−0.1706	0.5438	−0.4478	0.049*	
H8B	−0.2349	0.6781	−0.5163	0.049*	
C9	0.6850 (4)	0.8842 (4)	0.2928 (3)	0.0368 (5)	
O1	−0.3046 (2)	0.6962 (2)	−0.10853 (17)	0.0385 (4)	

### Atomic displacement parameters (Å^2^)

**Table:**

	*U*^11^	*U*^22^	*U*^33^	*U*^12^	*U*^13^	*U*^23^
Co1	0.0345 (3)	0.0389 (3)	0.0305 (3)	0.0226 (2)	0.0083 (2)	0.0097 (2)
S1	0.0535 (4)	0.0462 (4)	0.0441 (4)	0.0265 (4)	0.0163 (3)	0.0048 (3)
N1	0.0353 (10)	0.0408 (11)	0.0334 (10)	0.0222 (9)	0.0114 (8)	0.0142 (8)
N2	0.0369 (11)	0.0495 (12)	0.0346 (10)	0.0276 (10)	0.0121 (8)	0.0157 (9)
N3	0.0404 (11)	0.0456 (11)	0.0369 (11)	0.0262 (10)	0.0100 (9)	0.0114 (10)
C1	0.0401 (13)	0.0394 (12)	0.0372 (12)	0.0234 (11)	0.0121 (10)	0.0102 (10)
C2	0.0365 (12)	0.0426 (13)	0.0315 (12)	0.0212 (11)	0.0071 (10)	0.0082 (10)
C3	0.0384 (12)	0.0459 (13)	0.0300 (12)	0.0250 (11)	0.0141 (10)	0.0158 (10)
C4	0.0393 (13)	0.0399 (13)	0.0408 (13)	0.0225 (11)	0.0105 (10)	0.0149 (10)
C5	0.0352 (12)	0.0393 (13)	0.0403 (13)	0.0185 (11)	0.0083 (10)	0.0139 (10)
C6	0.0344 (12)	0.0361 (12)	0.0363 (12)	0.0186 (10)	0.0092 (10)	0.0124 (10)
C7	0.0438 (14)	0.0579 (15)	0.0361 (13)	0.0358 (13)	0.0143 (11)	0.0181 (11)
C8	0.0368 (12)	0.0589 (15)	0.0363 (13)	0.0308 (12)	0.0153 (10)	0.0207 (11)
C9	0.0322 (12)	0.0415 (13)	0.0374 (13)	0.0210 (11)	0.0109 (10)	0.0147 (11)
O1	0.0394 (9)	0.0435 (9)	0.0352 (9)	0.0253 (8)	0.0122 (7)	0.0124 (7)

### Geometric parameters (Å, °)

**Table:**

Co1—N1	2.1739 (19)	C2—H2	0.9300
Co1—N3	2.026 (2)	C3—C4	1.391 (3)
Co1—O1^i^	2.2034 (16)	C3—C6	1.496 (3)
S1—C9	1.620 (3)	C4—C5	1.372 (4)
N1—C1	1.343 (3)	C4—H4	0.9300
N1—C5	1.344 (3)	C5—H5	0.9300
N2—C6	1.331 (3)	C6—O1	1.256 (3)
N2—C7	1.464 (3)	C7—C8^ii^	1.515 (3)
N2—C8	1.475 (3)	C7—H7A	0.9700
N3—C9	1.170 (3)	C7—H7B	0.9700
C1—C2	1.377 (3)	C8—C7^ii^	1.515 (3)
C1—H1	0.9300	C8—H8A	0.9700
C2—C3	1.388 (3)	C8—H8B	0.9700
			
N3^iii^—Co1—N3	180.0	C3—C2—H2	120.3
N3^iii^—Co1—N1	90.06 (8)	C2—C3—C4	118.0 (2)
N3—Co1—N1	89.94 (8)	C2—C3—C6	121.7 (2)
N3^iii^—Co1—N1^iii^	89.94 (8)	C4—C3—C6	120.1 (2)
N3—Co1—N1^iii^	90.06 (8)	C5—C4—C3	118.8 (2)
N1—Co1—N1^iii^	180.00 (9)	C5—C4—H4	120.6
N3^iii^—Co1—O1^i^	90.43 (7)	C3—C4—H4	120.6
N3—Co1—O1^i^	89.57 (7)	N1—C5—C4	123.9 (2)
N1—Co1—O1^i^	89.79 (7)	N1—C5—H5	118.0
N1^iii^—Co1—O1^i^	90.21 (7)	C4—C5—H5	118.0
N3^iii^—Co1—O1^iv^	89.57 (7)	O1—C6—N2	122.7 (2)
N3—Co1—O1^iv^	90.43 (7)	O1—C6—C3	118.7 (2)
N1—Co1—O1^iv^	90.21 (7)	N2—C6—C3	118.7 (2)
N1^iii^—Co1—O1^iv^	89.79 (7)	N2—C7—C8^ii^	109.9 (2)
O1^i^—Co1—O1^iv^	180.00 (9)	N2—C7—H7A	109.7
C1—N1—C5	116.8 (2)	C8^ii^—C7—H7A	109.7
C1—N1—Co1	121.35 (15)	N2—C7—H7B	109.7
C5—N1—Co1	121.59 (15)	C8^ii^—C7—H7B	109.7
C6—N2—C7	121.13 (19)	H7A—C7—H7B	108.2
C6—N2—C8	125.8 (2)	N2—C8—C7^ii^	109.98 (19)
C7—N2—C8	112.94 (18)	N2—C8—H8A	109.7
C9—N3—Co1	171.96 (19)	C7^ii^—C8—H8A	109.7
N1—C1—C2	123.1 (2)	N2—C8—H8B	109.7
N1—C1—H1	118.4	C7^ii^—C8—H8B	109.7
C2—C1—H1	118.4	H8A—C8—H8B	108.2
C1—C2—C3	119.4 (2)	N3—C9—S1	179.0 (2)
C1—C2—H2	120.3	C6—O1—Co1^v^	127.28 (15)
			
N3^iii^—Co1—N1—C1	32.05 (18)	Co1—N1—C5—C4	−173.16 (18)
N3—Co1—N1—C1	−147.95 (18)	C3—C4—C5—N1	−0.4 (4)
O1^i^—Co1—N1—C1	−58.38 (17)	C7—N2—C6—O1	−1.9 (4)
O1^iv^—Co1—N1—C1	121.62 (17)	C8—N2—C6—O1	174.0 (2)
N3^iii^—Co1—N1—C5	−154.13 (18)	C7—N2—C6—C3	178.1 (2)
N3—Co1—N1—C5	25.87 (18)	C8—N2—C6—C3	−6.1 (3)
O1^i^—Co1—N1—C5	115.44 (18)	C2—C3—C6—O1	110.4 (3)
O1^iv^—Co1—N1—C5	−64.56 (18)	C4—C3—C6—O1	−64.0 (3)
C5—N1—C1—C2	−0.9 (3)	C2—C3—C6—N2	−69.5 (3)
Co1—N1—C1—C2	173.23 (17)	C4—C3—C6—N2	116.0 (3)
N1—C1—C2—C3	0.3 (4)	C6—N2—C7—C8^ii^	−126.9 (2)
C1—C2—C3—C4	0.2 (3)	C8—N2—C7—C8^ii^	56.8 (3)
C1—C2—C3—C6	−174.4 (2)	C6—N2—C8—C7^ii^	127.0 (2)
C2—C3—C4—C5	−0.2 (3)	C7—N2—C8—C7^ii^	−56.8 (3)
C6—C3—C4—C5	174.5 (2)	N2—C6—O1—Co1^v^	100.8 (2)
C1—N1—C5—C4	0.9 (3)	C3—C6—O1—Co1^v^	−79.1 (2)

Symmetry codes: (i) −*x*, −*y*+1, −*z*; (ii) −*x*−1, −*y*+1, −*z*−1; (iii) −*x*+1, −*y*+1, −*z*; (iv) *x*+1, *y*, *z*; (v) *x*−1, *y*, *z*.
